# Surgical correction of severe rigid equinovarus deformity in Cockayne syndrome: perioperative challenges and multidisciplinary management—a case report

**DOI:** 10.1093/jscr/rjag732

**Published:** 2026-08-23

**Authors:** Shadi Abu Isneina, Bessan Hamed Dababseh, Ala’a S Ghnimat, Lubna W AbuHamdiya, Dina Wohoosh, Abdallah Dababsseh

**Affiliations:** Orthopaedics Department, Palestine Polytechnic University, Wadi Al Haria, PO Box 198, Hebron, Palestine; Department of Clinical Medical Sciences, Faculty of Medicine and Health Sciences, Palestine Polytechnic University, Wadi Al Haria, PO Box 198, Hebron, Palestine; Department of Clinical Medical Sciences, Faculty of Medicine and Health Sciences, Palestine Polytechnic University, Wadi Al Haria, PO Box 198, Hebron, Palestine; Department of Clinical Medical Sciences, Faculty of Medicine and Health Sciences, Palestine Polytechnic University, Wadi Al Haria, PO Box 198, Hebron, Palestine; Department of Clinical Medical Sciences, Faculty of Medicine and Health Sciences, Palestine Polytechnic University, Wadi Al Haria, PO Box 198, Hebron, Palestine; Department of Clinical Medical Sciences, Faculty of Medicine, Alexandria University, 22 El-Guish Road, El-Shatby, Alexandria 21526, Egypt

**Keywords:** Cockayne syndrome, equinovarus deformity, Achilles tendon lengthening, perioperative management, *ERCC6,* DNA repair disorder, pediatric orthopedics

## Abstract

Cockayne syndrome (CS) is a rare autosomal recessive transcription-coupled nucleotide excision repair disorder (*ERCC6*/*ERCC8*) causing progressive neuromuscular deterioration and limb contractures. We report a 10-year-old boy with CS type B (*ERCC6*) who presented with rigid bilateral equinovarus, rendering him non-ambulatory, and elevated transaminases (ALT 682 U/L, AST 183 U/L). Bilateral fractional elongation of the Achilles tendon was performed successfully; hepatotoxic agents were avoided, and opioid/sedative dosages were meticulously titrated. Recovery was uneventful; the patient regained supported standing and resumed active physiotherapy. Functional orthopedic surgery is achievable in CS via rigorous multidisciplinary coordination, providing a valuable template for perioperative risk management.

## Introduction

Cockayne syndrome (CS) is a rare progeroid disorder caused by biallelic pathogenic variants in *ERCC6* or *ERCC8*, impairing transcription-coupled nucleotide excision repair (TC-NER) [1, 2]. Its clinical triad of postnatal growth failure, progressive neurodegeneration, and cutaneous photosensitivity is accompanied by broader multisystem involvement, including hearing loss, retinal dystrophy, dental anomalies, and hepatic dysfunction [1, 3].

Progressive spasticity and leukodystrophy in CS frequently produce fixed lower-limb contractures [4]. Severe rigid equinovarus deformity, driven by Achilles tendon contracture, is a debilitating yet poorly characterized complication causing intractable pain, loss of ambulation, and orthotic intolerance [4, 5]. The orthopedic literature offers little guidance on the surgical correction of these deformities in CS.

Surgical decision-making is complicated by the systemic fragility inherent to CS, including heightened anesthetic sensitivity, potential hepatotoxicity, and the syndrome’s progressive, fatal course [6]. We report a 10-year-old boy with CS type B and compound heterozygous *ERCC6* mutations who underwent successful bilateral fractional elongation of the Achilles tendon (ETA) despite markedly elevated hepatic transaminases, aiming to describe a reproducible surgical technique, a perioperative framework for multisystem fragility, and the role of multidisciplinary care, including genetic counseling [1, 3, 6].

## Case presentation

### History and diagnosis

A 10-year-old boy of non-consanguineous parentage presented with a two-year history of progressive gait deterioration. Cutaneous photosensitivity noted at four months of age prompted genetic evaluation and a diagnosis of CS. A sister with the same diagnosis died at age five from leukemia-associated complications, consistent with the genomic instability reported with specific *ERCC6* variants [3, 7].

### Genetic analysis

Molecular testing identified two pathogenic *ERCC6* variants, one inherited from each parent, confirming a compound heterozygous state consistent with CS type B. Two siblings were confirmed heterozygous carriers; one carried wild-type alleles at both loci [3].

### Clinical progression and examination

**Figure 1:**
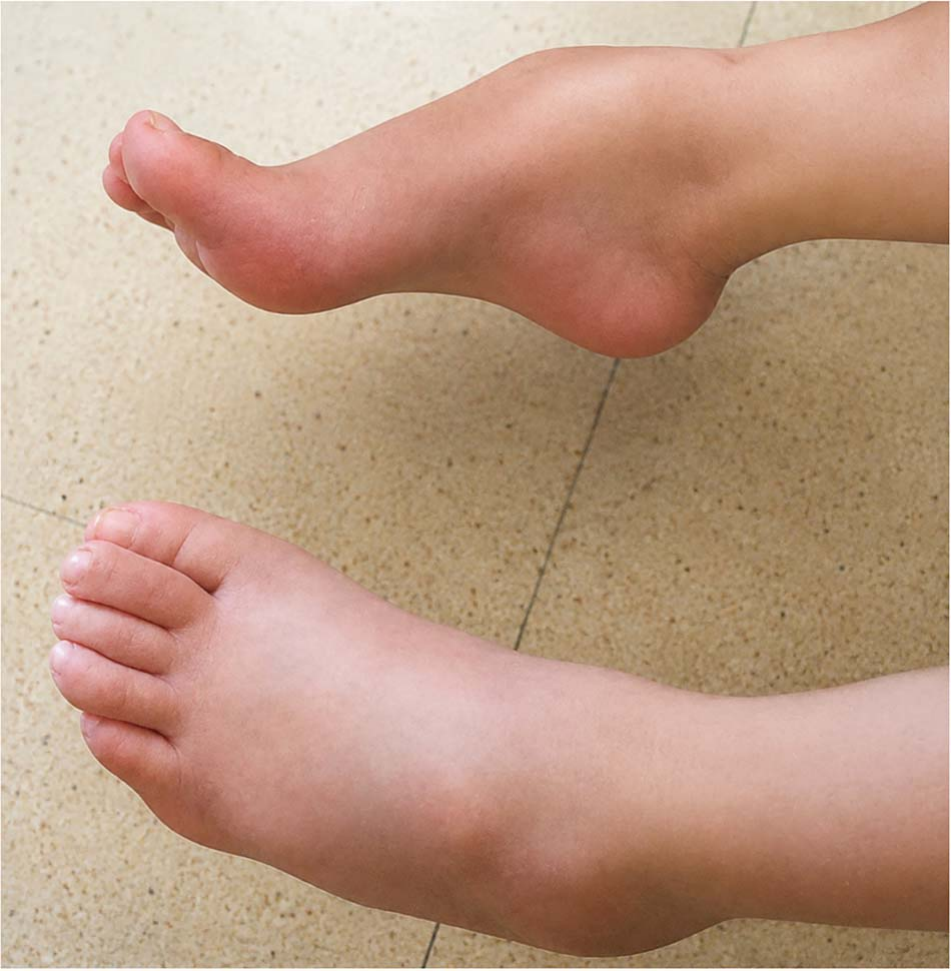
Severe rigid bilateral equinovarus deformity at presentation, demonstrating marked plantar flexion, hindfoot varus, and forefoot adduction of both feet.

At age eight, the patient developed a waddling gait; physiotherapy compliance was inconsistent, and progressive spasticity culminated in the complete loss of ambulation by age ten, with severe rigid bilateral equinovarus deformity (Fig. 1). Anthropometric parameters were below the third percentile, consistent with the growth failure and microcephaly characteristic of CS [1]. Findings included dysmorphic facial features, bilateral Achilles tendon rigidity, spasticity with hyperreflexia, mild axial hypotonia, a hand tremor, and intellectual disability. Gastrointestinal symptoms included constipation, abdominal pain, and oropharyngeal dysphagia.

### Perioperative hepatic concern

Preoperative biochemistry revealed markedly elevated liver transaminases (ALT 682 U/L; AST 183 U/L), a recognized but infrequently detailed complication of CS attributed to impaired TC-NER in hepatocytes [6]. This prompted a formal multidisciplinary review—hepatology, anesthesiology, and orthopedic surgery—to stratify perioperative hepatic risk.

### Surgical procedure and perioperative management

Surgery was indicated given the severity and rigidity of the deformity, the failure of a two-year course of physiotherapy, and the patient’s inability to tolerate orthotic devices. Following multidisciplinary consensus, the patient underwent bilateral fractional ETA. Hepatotoxic agents, including metronidazole, were strictly avoided; volatile anesthetic agents with favorable hepatic safety profiles were selected; and sedative and opioid doses were titrated conservatively with enhanced monitoring [6]. Adequate elongation was confirmed intraoperatively by achieving passive ankle dorsiflexion to neutral with the knee in full extension. The intraoperative and postoperative course was uneventful, and the patient was discharged the following day with oral analgesics and prophylactic antibiotics.

### Post-operative management and outcome

The patient was immobilized in bilateral below-knee casts for six weeks, then transitioned to bilateral ankle-foot orthoses to maintain correction and support rehabilitation. At the six-month follow-up, the patient was able to stand with support and remained engaged in structured physiotherapy; independent ambulation had not yet been achieved but remained the ongoing rehabilitation goal.

## Discussion

This report describes the first detailed account of the surgical correction of a severe rigid bilateral equinovarus deformity in CS type B with concomitant hepatic dysfunction, providing a reproducible perioperative framework for orthopedic surgeons.

The equinovarus deformity in CS is not a primary skeletal malformation but a secondary consequence of progressive spasticity and leukodystrophy [4, 8]. Demyelination and axonal loss in the corticospinal tracts produce a chronic upper motor neuron pattern of neuromuscular imbalance, with the triceps surae overpowering its antagonists, producing a fixed Achilles tendon contracture. This complication is likely underreported in CS [4].

Fractional ETA is the standard of care for fixed equinus deformity in neurologically impaired children when conservative measures fail [9, 10], restoring the dorsiflexion range, correcting gait biomechanics, and permitting orthotic fitting [10]. In our patient, a two-year trial of physiotherapy produced no improvement and the contracture precluded orthotic tolerance, making surgery both indicated and urgent.

The markedly elevated transaminases represented the principal perioperative risk, likely reflecting accumulated oxidative DNA damage in metabolically active hepatocytes [6, 7]. Khawaja and Tobias previously reported the perioperative management of a CS patient undergoing bilateral clubfoot correction, noting a heightened risk of anesthetic-related hepatotoxicity [6]. Our case extends this experience, showing that safe surgical correction is achievable even with substantially elevated transaminases when hepatotoxic agents are avoided, anesthetic dosing is conservatively titrated, and decisions follow a formal multidisciplinary review. Al Kaissi *et al*. [4] similarly noted that orthopedic deformities in CS may be underrecognized; our findings extend this to an older, more compromised patient.

Genetic counseling is integral to care: the delineation of mutations, carrier status, and genotype–phenotype correlations—illustrated by the sister’s leukemia-associated death—informs realistic family expectations [3, 7]. Continuous orthopedic surveillance is warranted, as recurrent contractures and new joint deformities are expected even after successful surgery [1].

The principal limitations of this report are its single-case design and the absence of long-term follow-up beyond six months; multi-center case series are needed to establish evidence-based orthopedic management guidelines for CS.

## Conclusion

Severe rigid equinovarus deformity is a clinically significant, under-recognized complication of CS that can render affected children non-ambulatory. This case demonstrates that safe, effective surgical correction via bilateral fractional Achilles tendon elongation is achievable even with significant hepatic dysfunction, provided management follows a structured multidisciplinary perioperative strategy with the strict avoidance of hepatotoxic agents and conservative titration of anesthetic and analgesic drugs. This report offers a practical clinical template for orthopedic surgeons and a foundation for future collaborative studies aimed at evidence-based surgical guidelines for this vulnerable population.

## Data Availability

All data supporting the conclusions of this report are contained within the article. Additional data are available from the corresponding author upon reasonable request.
